# Contactless Measurement of Magnetic Nanoparticles on Lateral Flow Strips Using Tunneling Magnetoresistance (TMR) Sensors in Differential Configuration

**DOI:** 10.3390/s16122130

**Published:** 2016-12-14

**Authors:** Huaming Lei, Kan Wang, Xiaojun Ji, Daxiang Cui

**Affiliations:** 1 Department of Instrument Science and Engineering, Shanghai Jiaotong University, Shanghai 200240, China; jxj127@sjtu.edu.cn (X.J.); dxcui@sjtu.edu.cn (D.C.); 2 Shanghai Engineering Research Center for Intelligent Diagnosis and Treatment Instrument, Shanghai 200240, China

**Keywords:** magnetic nanoparticles, tunneling magnetoresistance (TMR) sensor, differential sensing, lateral flow strip (LFS), magnetic biosensor

## Abstract

Magnetic nanoparticles (MNPs) are commonly used in biomedical detection due to their capability to bind with some specific antibodies. Quantification of biological entities could be realized by measuring the magnetic response of MNPs after the binding process. This paper presents a contactless scanning prototype based on tunneling magnetoresistance (TMR) sensors for quantification of MNPs present in lateral flow strips (LFSs). The sensing unit of the prototype composes of two active TMR elements, which are parallel and closely arranged to form a differential sensing configuration in a perpendicular magnetic field. Geometrical parameters of the configuration are optimized according to theoretical analysis of the stray magnetic field produced by the test line (T-line) while strips being scanned. A brief description of our prototype and the sample preparation is presented. Experimental results show that the prototype exhibits the performance of high sensitivity and strong anti-interference ability. Meanwhile, the detection speed has been improved compared with existing similar techniques. The proposed prototype demonstrates a good sensitivity for detecting samples containing human chorionic gonadotropin (hCG) at a concentration of 25 mIU/mL. The T-line produced by the sample with low concentration is almost beyond the visual limit and produces a maximum stray magnetic field some 0.247 mOe at the sensor in the *x* direction.

## 1. Introduction

**Figure 1:**
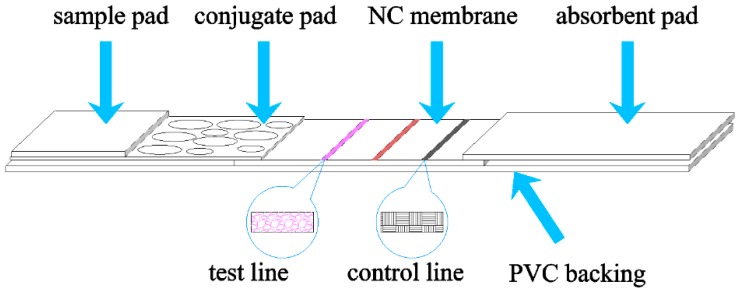
Structure of a lateral flow strip. It has at least one test line and a control line which serves as the reaction area.

Magnetic nanoparticles (MNPs) are commonly used as labels in biomedical application [1,2,3,4]. Lateral flow strips (LFSs) with MNPs have been developed as new tools for sensitive diagnostic applications owing to their capability to bind with some specific antibodies. Quantification of biological entities could be realized by measuring the magnetic response of MNPs after the binding process [5,6]. The strip usually includes five parts, as shown in Figure 1. The sample pad is an absorbent pad onto which the test sample is applied. The conjugate pad contains antibodies specific to the target analyte which bind to labeling nanoparticles and make them release stably. A nitrocellulose (NC) membrane provides the reaction area including at least one test line (T-line) and a control line (C-line). These lines cross the membrane to act as a capture zone for antibodies immobilization. The absorbent pad provides the driving force to draw the sample across the reaction membrane by capillary action and to collect it. In addition, a polyvinyl chloride (PVC) backing plate is used as support.

Conventional tests based on immune recognition and the use of colored colloidal particles have still some drawbacks that limit their use: they do not provide a quantitative determination of the analyte, and their sensitivity is limited [7]. To overcome the disadvantages, inductive techniques [8,9,10,11,12] and magnetic sensors, such as giant magnetoresistance (GMR) [7,13,14,15,16,17] sensors and anisotropic magnetoresistance (AMR) sensors [18] have been used to successfully quantify the nanoparticles by measuring the magnetic response of MNPs.

Unfortunately, many GMR sensor-based systems are realized by an on-chip method because they have to maintain a small liftoff between the sensing element and MNPs due to their limited sensitivity and considerable noise. This makes them difficult to realize as non-contact tests. Usually, the sensors of such systems can be utilized only once or they need to undergo a complicated process for reuse. In examples from the literature [19,20], GMR biosensor chips were triple washed with acetone, methanol, and isopropyl alcohol solution, then blow dried. The chips were exposed to oxygen plasma for 1 min to remove remaining organic contaminants. Obviously, the cost will increase and they become inconvenient to use. Moreover, the spin valve (SV) in GMR sensors designed for biodetection might be in contact with some chemicals in the biomolecular medium which protects the biomolecules but may harm the SV. Sometimes this also results in contact noise. Certainly, there are also some systems suitable for contactless or wash-free measurement [7,21]. In contactless systems, the GMR sensor and the tested sample are separated and kept at a small but stable lift-off. Otherwise, the signal will suffer a dramatic decay as the lift-off increases. Generally, GMR sensors are more attractive as these biosensor systems exhibit several technical advantages such as lower power consumption, better portability, and less complexity when compared with commercial systems based on inductive techniques.

Recently, tunneling magnetoresistance (TMR) sensors which have a much higher magnetoresistance ratio (MR ratio) than GMR were introduced to meet the challenges of biosensing [22,23]. TMR spin-valves operate based on a quantum mechanical effect known as spin-dependent tunneling. This effect will change the resistance of the sensor as a function of the external magnetic field. By monitoring resistance changes caused by the stray magnetic field from MNPs, a quantitative platform could be created to accurately determine the amount of biomarkers captured. The most significant difference between TMR and GMR is that the spacer layer between two magnetic layers is changed from a conductive metal layer, such as copper, for GMR, to a nonconductive barrier layer, e.g., aluminum oxide, for TMR. This makes TMR have a much higher sensitivity. Sensors with MR ratios of up to 300% are available in commercial products. Other favorable features of TMR sensors include low field-noise and small active areas. These factors guarantee the high performance of superior sensitivity and high spatial resolution for low-field measurement.

In this paper, a theoretical analysis of the stray magnetic field as well as the differential sensing method is discussed in Section 2. Analytical solution of stray magnetic field produced by T-line when the strips being tested in a perpendicular magnetic field is achieved by the approximation with line magnetic dipole model. Differential sensing configuration is optimized according to features of the stray magnetic field. An experimental prototype based on Wheatstone bridge type TMR sensors, as well as sample preparation is described in Section 3. Section 4 shows the experimental results of measuring samples with a concentration gradient of containing 25, 50, 100, 200, 400 and 800 mIU/mL of human chorionic gonadotropin (hCG). System sensitivity and anti-interference ability based on the proposed configuration are evaluated and discussed.

## 2. Theory and Methods

### 2.1. Analytical Signal Produced by Magnetic Strips

**Figure 2:**
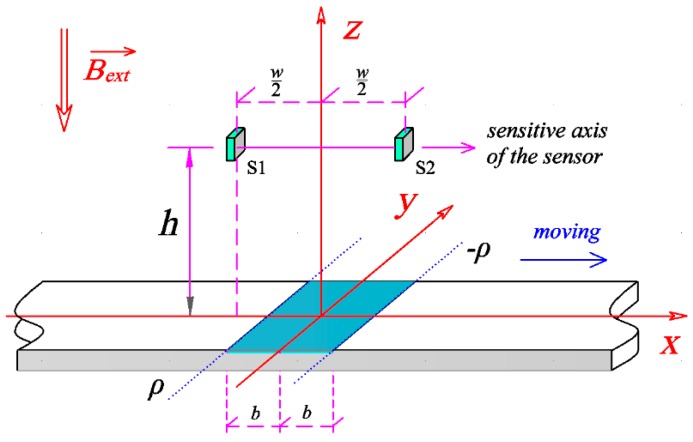
Measurement configuration with two active sensors S1 and S2. The two sensors locate at a liftoff *h* and with *a* gap *w*. The external magnetic induction is perpendicular to the strip surface.

The detection of MNPs depends on the application of an external magnetic field, $\vec{H_{ext}}$, or an external magnetic induction, $\vec{B_{ext}}$, as excitation. The measurement configuration is sketched in Figure 2. It is fairly assumed that there is a uniform distribution of particles immobilized on the MNPs layer and the thickness of the layer is much smaller than the width and length. The external magnetic induction, $\vec{B_{ext}}$, is perpendicular to the surface of the strips. Since the test area, also known as test line, usually appears as an elongated rectangle, the length of the test line in *y*-axis is several times larger than its width 2*b* in the *x*-axis. To analyze a 2-D magnetic field in the center plane *x*-*z*, the stray magnetic field produced by test line can be approximately predicted by the line magnetic dipole model as the one used so far for nondestructive testing (NDT) [24,25].

In our idealized model, we assume that the line magnetic dipole composes of two infinitely long lines with equal but opposite magnetic charge. The two lines are parallel to each other with a gap of *2b*. The dipole moment of the lines is ρ per unit length in the *y* direction. Considering the complex field at a spatial point (*x*, *z*) out of the test line, the synthetic magnetic field produced by the two magnetic lines is denoted as:

(1) $$H=H_{x}\left(x,z\right)\hat{x}+H_{z}\left(x,z\right)\hat{z}$$

The *x*-axis and *z*-axis components of the stray magnetic field can be inferred as:

(2) $$H_{x}\left(x,z\right)=-\frac{2\rho bxz}{\left[{\left(x+b\right)}^{2}+z^{2}\right]\left[{\left(x-b\right)}^{2}+z^{2}\right]}$$

(3) $$H_{z}\left(x,z\right)=-\frac{\rho \left(x^{2}-z^{2}-b^{2}\right)}{\left[{\left(x+b\right)}^{2}+z^{2}\right]\left[{\left(x-b\right)}^{2}+z^{2}\right]}$$

**Figure 3:**
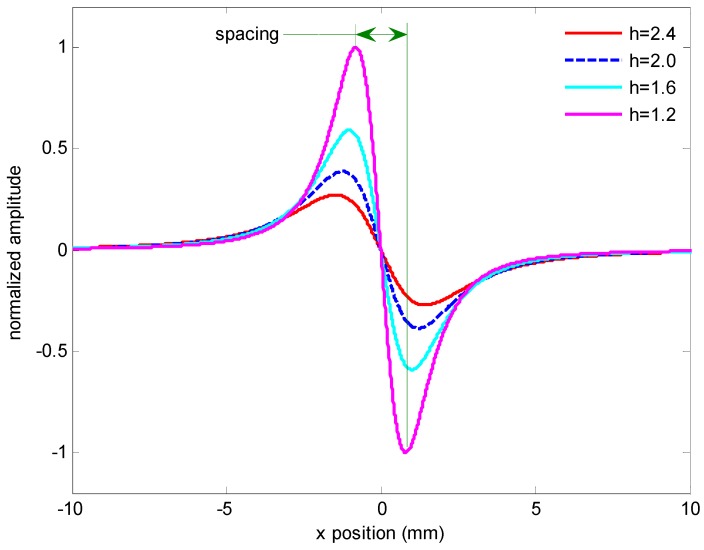
Typical signals *H_x_* predicted by line magnetic dipole model.

The dipole moment ρ is a function of $\vec{B_{ext}}$, written as $\rho =\chi B_{ext}$, and *χ* is the MNPs volume magnetic susceptibility. The external magnetic induction, $\vec{B_{ext}}$, is perpendicular to the sensitive axis of the sensor in order to prevent saturation and enable high dynamic range measurements. Therefore, the *x*-axis component is studied for the measurement. It usually takes the shape as shown in Figure 3, as an example, with the test line width 1 mm and a liftoff 1.2, 1.6, 2.0 and 2.4 mm, respectively. The signals are normalized by the one with a liftoff of 1.2 mm. Obviously, with the increasing liftoff, the amplitude decreases and the spacing between the positive peak and the negative peak increases. These features are useful for developing a differential sensing prototype.

Although these signals are derived from the idealized model, they are similar to those presented in the literature [23,26]. A 2-D analytical investigation is also developed to verify Equation (2) by the method described by Fodil [27]. The same expression for *H_x_* is obtained when we assume that the line length is infinitely long. In particular, we focus more on the waveform of the signal than on the value itself when optimizing the sensing configuration. When the idealized model is compared with the analytical one with finite geometry, for example, for a T-line with a width 1.0 mm and length 3.0 mm, the difference of the spacing between the positive peak and the negative peak does not exceed 0.12 mm as the liftoff varies from 1.6 mm to 2.5 mm. This ensures the effectiveness of the method described below.

### 2.2. Differential Sensing Method

Taking into account the anti-symmetry of the *x* component as described in Equation (2) and shown in Figure 3, a differential sensing configuration is proposed for the detection of the weak signal *H_x_* as shown in Figure 2. Two magnetic sensors S1 and S2 are the Wheatstone bridge TMR sensors and located at point (−*w/*2, 0, *h*) and (*w/*2, 0, *h*), respectively. Here *w* is the gap between the two sensors and *h* is the lift off. The external magnetic induction is perpendicular to the surface of the test line and the sensitive axis of the sensor.

We consider a strategy that uses the difference of two sensors’ outputs to improve the quality of the signal. The two magnetic sensors are in close proximity. When there is outside interference applied to the devices, the interference can be regarded as a common mode signal because the interference acting on the two sensors is almost identical. The outputs cancel each other due to the differential effects. However, the signal generated by the test line is antisymmetric. It will generate the opposite effects on the two sensors when the signal is applied to the device. The output signal will be strengthened when the gap *w* between S1 and S2 has an optimized dimension. As the test strip moves along the *x* direction, the stray magnetic field in *x-*axis *H_x_* generated by the T-line at points S1 and S2 can be written as:

(4) $$H_{xs1}=-\frac{\rho b\left(x+\frac{w}{2}\right)h}{\left[{\left(x+\frac{w}{2}+b\right)}^{2}+h^{2}\right]\left[{\left(x+\frac{w}{2}-b\right)}^{2}+h^{2}\right]}$$

(5) $$H_{xs2}=-\frac{\rho b\left(x-\frac{w}{2}\right)h}{\left[{\left(x-\frac{w}{2}+b\right)}^{2}+h^{2}\right]\left[{\left(x-\frac{w}{2}-b\right)}^{2}+h^{2}\right]}$$

**Figure 4:**
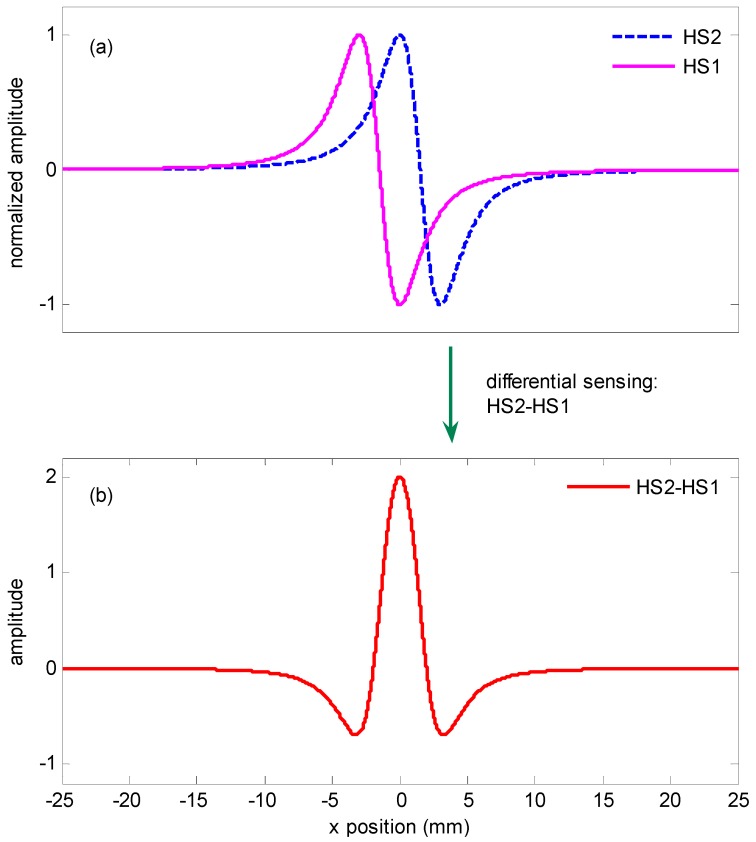
Signals of differential sensing strategy. (**a**) normalized signals on each sensors with a liftoff 2.5 mm and test line width 1 mm; (**b**) differential signal $H_{s2}-H_{s1}$.

The differential output of the two elements is expressed as Equation (6), and the normalized signal is shown in Figure 4. Figure 4a shows the predicted signals at sensors S1 and S2 with a liftoff *h =* 2.5 mm and test line width 2*b =* 1 mm. Figure 4b is the differential output of the two signals:

(6) $$H_{x△}=H_{xs2}-H_{xs1}$$

If the derivative of Equation (6) with respect to *x* is set to zero, i.e., $dH_{x△}/dx=0$, the differential signal $H_{x△}$ will have an extreme value which can be expressed as:

(7) $$H_{x△max}=\frac{\rho bwh}{\left[{\left(\frac{w}{2}+b\right)}^{2}+h^{2}\right]\left[{\left(\frac{w}{2}-b\right)}^{2}+h^{2}\right]}$$

In order to optimize the configuration of the differential sensing structure, the spacing *w* between S1 and S2 is deduced by assuming $dH_{x△max}/dx=0$, and we get:

(8) $$w_{gap}=2\sqrt{\frac{b^{2}-h^{2}+2\sqrt{b^{4}+h^{4}+b^{2}h^{2}}}{3}}$$

The liftoff *h* and the test line width 2*b* are determined by actual conditions. If the gap *w* takes a value as described in Equation (8), the differential signal will be the strongest at a specific liftoff *h*. The maximum amplitude of the differential signal will change as the gap *w* varies. It is worth noting that the liftoff distance plays a key role on signal amplitude. The smaller the lift-off is, the stronger the signal will be. Nevertheless, in order to meet the needs of non-contact detection, usually it is necessary to maintain the liftoff at a certain distance. The detection will achieve the best signal by optimizing the gap *w* under a specified lift-off value. In the following experiments, a contactless measurement is performed and the configuration is optimized according to Equation (8).

## 3. Experiments

### 3.1. Experimental Prototype

**Figure 5:**
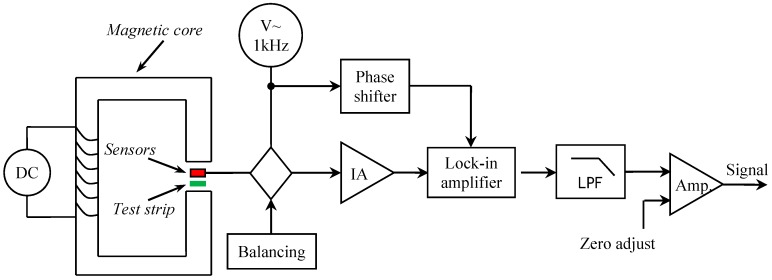
Schematic diagram of LFSs detection system based on differential configuration.

A schematic diagram of the experimental prototype can be viewed in Figure 5. Briefly, the standard immunoassay test is performed as follows: the test strip is moving along the *x* direction as shown in Figure 2 and kept in a stable liftoff of 2.5 mm by a mechanical system. Stray magnetic field arising from the MNPs on the test line is detected by the aforementioned TMR sensors in differential configuration with a gap of 3 mm as optimized. Both TMR sensors are active in sensing the stray magnetic field because they are equivalently close to the test trip. The differential measurement cancels out most of the influence of homogeneous external magnetic fields such as earth magnetic field or power frequency interference due to their proximate arrangement. On the other hand, it doubles the target signal compared with that in the case of using only one active sensing element. This is different from the method in which one sensor is used as an active sensor for sensing the stray magnetic field and another is used as passive one for cancelling out the interference.

Bridge circuits that utilize DC excitation are often plagued by errors caused by thermocouple effects, 1/f noise, DC drifts in the electronics, and line noise pick-up. One way to get around these problems is to excite the bridge with an AC waveform, amplify the bridge output with an AC amplifier, and synchronously demodulate the resulting signal. An AC excitation with frequency 1 kHz and amplitude no larger than 6 Vpp is applied to the TMR sensors, to avoid the sensor heating. The AC phase and amplitude information from the bridge are recovered to become a DC signal at the output of the synchronous demodulator. Thus, very slight change in the magnetic field caused by the stray magnetic field could be detected with a high interference suppression.

**Figure 6:**
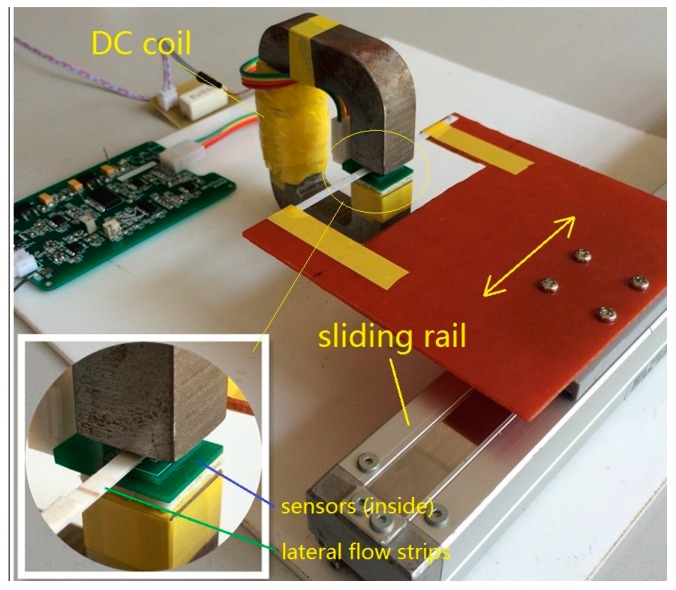
General view of the prototype. The sensors are TMR2705 and the magnetic core is with a cross-section 16 × 16 mm and an air gap 8 mm.

DC current is applied to magnetize the C-shaped magnetic core to provide a magnetic field perpendicular to the test strip about 300 Oe and with a field homogeneity of less than 7% in the most center effective area. Here the magnetic field homogeneity is defined as ∆B/B in the most center 25% area of the cross-section. Cross-section dimension and an air gap in the C-shaped magnetic core is optimized to ensure the homogeneity of the magnetic field. The C-shaped magnetic core is developed with a cross-section 16 × 16 mm^2^ and an air gap 8 mm. The prototype is illustrated in Figure 6. In the experiments, the sensors are sensitive to the magnetic field in *x* direction and their model is TMR2705, which are manufactured by Dowaytech Co, Ltd., (Suzhou, China). The LFS is moved along the *x* direction through a sliding rail controlled by a step motor.

### 3.2. Sample Preparation

Hydrophilic superparamagnetic nanoparticles with carboxyl groups were prepared by our group members using a modified one-step hydrothermal synthesis [28]. Details of the MNP fabrication were published elsewhere [29]. The hydrodynamic size of MNPs is 80 nm. Both antigen and antibody of hCG were purchased from Shanghai Linc Bio Science Co. (Shanghai, China). Additional materials, including conjugate pads, sample pads, absorbent pads, NC membrane and PVC plate were purchased from JieYi Biotech Co., Ltd. (Shanghai, China).

Monoclonal antibodies β (mAb2) and second antibody (goat-anti-mouse, IgG) were coated separately to serve as T-line and C-line. Both antibodies were dispensed to NC membrane while the MNPs were dispensed onto the conjugate pad. After being dried at 37 °C, all the pretreated parts were pasted in a certain order on a PVC backing plate with 2 mm overlap of each component. The assembly was cut into 3 mm wide individual strips and then stored with a desiccant at room temperature inside a sealed plastic container until use.

**Figure 7:**
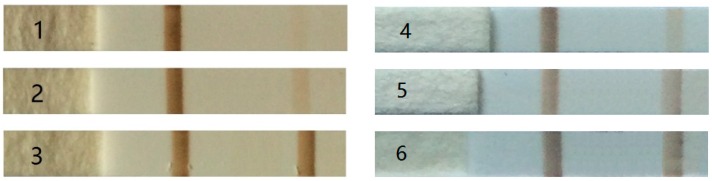
Standard lateral flow strips for sensitivity and limit test with different hCG concentration. 1–25 mIU/mL, 2–50 mIU/mL, 3–800 mIU/mL, 4–100 mIU/mL, 5–200 mIU/mL, 6–400 mIU/mL.

To evaluate the detection sensitivity of our prototype, a standard curve is generated by detecting standard hCG antigen solutions at a concentration gradient of 25, 50, 100, 200, 400, and 800 mIU/mL diluted in ultrapure water (1 IU of hCG is equal to 0.11 ug of hormone). Samples for sensitivity test are shown in Figure 7. In order to reduce cross interference of the stray magnetic field produced by T-line and C-line, the distance between the two lines is some 10 mm in the first step of our study. The stray magnetic intensity produced by T-line and concentration of hCG antigen in the sample is in a direct ratio. Analyzing the variation tendency of T/C ratio (ratio between T-line and C-line signal intensity) we could draw a standard curve and estimate the limit of detection.

## 4. Results and Discussion

**Figure 8:**
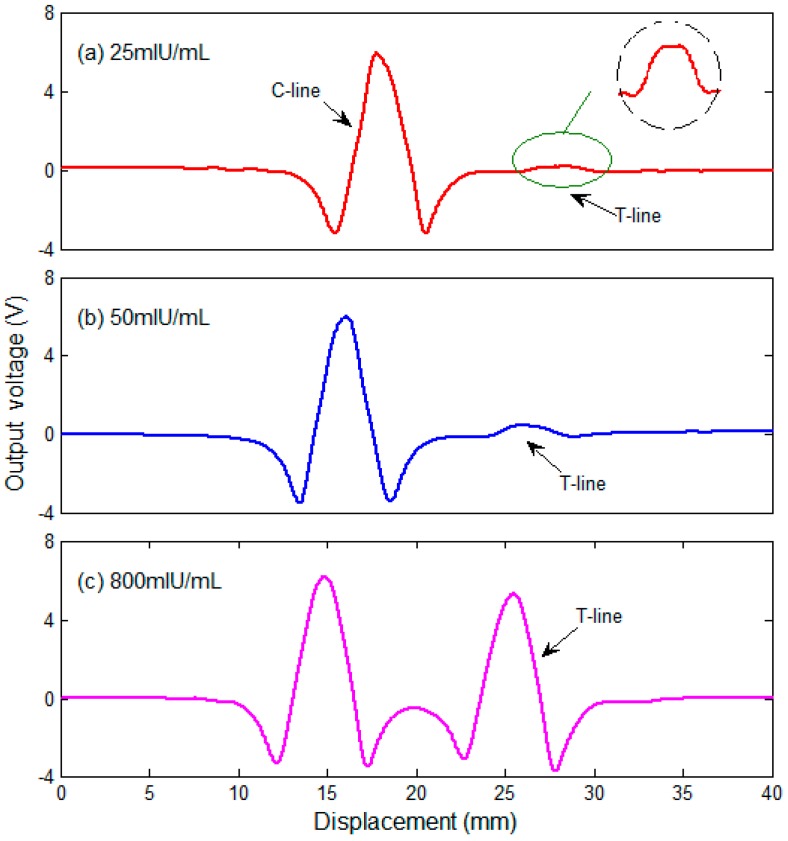
Output signals of measurement corresponding to samples containing (**a**) 25 mIU/mL; (**b**) 50 mIU/mL; and (**c**) 800 mIU/mL of hCG.

Experiments were conducted to evaluate the performance of the experimental prototype. Samples containing hCG with a concentration gradient of 25, 50, 100, 200, 400 and 800 mIU/mL were tested in the experiments. A set of output signals at a low concentration of 25, 50 and a high concentration of 800 mIU/mL is shown in Figure 8. The shape of the signal fits well with theoretical analysis of the stray magnetic field produced by the MNPs. The prototype is able to sense a sample as low as 25 mIU/mL whose T-line is almost beyond the visual limit as shown in Figure 7. The concentration is calculated based on the voltage caused by T-line by comparing with that caused by C-line. The test results for the three samples are 23.61, 50.02, 747.24 mIU/mL, respectively, which are very close to the nominal value. It is worth to note a detail that the two negative peaks caused by the T-line shown in Figure 8c do not exhibit the same height. Through careful observation of the samples shown in Figure 7, this phenomenon might be caused by the uneven distribution of particles within the T-line.

Measurement of a sample containing 25 mIU/mL of hCG shows that the prototype produced an output voltage of 157.8 mV at the peak. Taking into account the sensor sensitivity and the gain of the detection system, the output voltage indicates that the T-line produces a maximum magnetic field in the *x* direction of 0.247 mOe at the sensor. This indicates that the detection limit of the system is better than 0.247 mOe. It should be noted that we use the minimum detectable magnetic field, rather than the concentration of solution itself as the detection limit of the system. As we know, many factors, such as particles diameter, magnetization intensity and liftoff, will directly affect the intensity of the stray magnetic field. For example, the magnetic moment of a spherical MNP is proportional to the 3rd power of its diameter [10]. As a comparison, the diameter of MNPs used in literature [7] is 184 ± 4.5 nm and a stray magnetic field with an intensity of 0.19 Oe is observed by GMR sensors. While the diameter of MNPs used in our experiments is about 80 nm, and the stray magnetic field is much weaker. That is 0.247 mOe.

**Figure 9:**
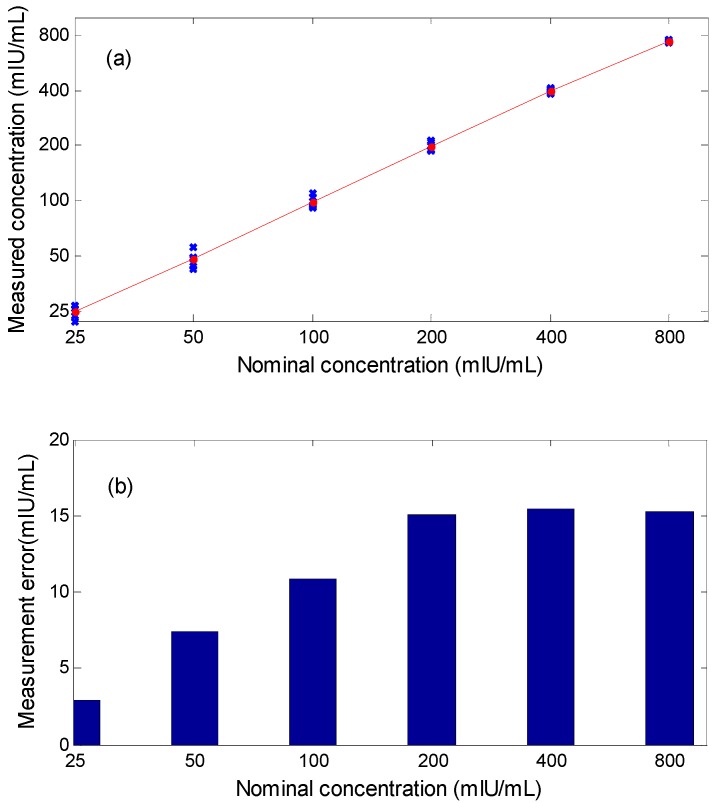
Experimental results for the measurement of standard strips. (**a**) measured concentration vs. nominal concentration. X-crosses: result of one single measurement; dots: average of five measurements; (**b**) measurement error.

In Figure 9a, the results of measuring the standard strips are shown. X-crosses correspond to one single measurement and dots correspond to the average of five measurements. The measured concentration is calculated based on the T/C ratio of the output voltage. As expected, it shows that the signal arising from the sensors is linear with the concentration. According to the results depicted in Figure 8 and Figure 9, our prototype has a sensitivity of 11.9 mV/(mIU/mL). Maximum measurement errors to their average values are 2.9, 7.4, 10.9, 15.1, 15.5 and 15.3 mIU/mL, respectively, at 25, 50, 100, 200, 400 and 800 mIU/mL correspondingly. The error bars are shown in Figure 9b. Predictably, a portion of the error is due to analog-digital convertor (ADC) while the voltage range for sampling varies. The accuracy of standard test strips themselves is also an important factor which influences the calibration results. Another point which is worth to mention is that it only requires six seconds in our experiments to fulfill a single scanning. This greatly speeds up the detection efficiency compared with existing similar techniques. It took 5 min to complete a scan in the literature [7] and about 20 min for the magnetic immuno-chromatographic test (MICT) system provided by MagnaBiosciences, Inc. (San Diego, CA, USA) [30,31].

## 5. Conclusions and Perspectives

A theoretical analysis of the stray magnetic field produced by a test strip as well as a differential sensing method has been presented. An experimental prototype has been developed to fulfill the contactless measurement of nanomagnetic LFSs. Comparing the experimental results with our theoretical analysis leads to the conclusion that the experimental prototype using TMR sensors in differential configuration can effectively suppress the noise and improve the sensitivity and limit of detection. A signal with high quality is achieved. By adopting a lock-in amplifier and demodulating techniques, interference has been significantly suppressed and the measurement time has been greatly reduced to six seconds. This is very competitive compared with existing similar techniques.

To achieve a multi-target detection, further research will be focused on analysis and characterization of the signal crosstalk as there is a plurality of test lines on a test strip. Effects and measurement of uneven distribution of magnetic nanoparticles on the T-line also will be studied. Further efforts will be made to make the platform both cost effective and power efficient for portable biosensing applications.
